# Comparative proteomic analysis of the ovarian fluid and eggs of Siberian sturgeon

**DOI:** 10.1186/s12864-024-10309-y

**Published:** 2024-05-07

**Authors:** Natalia Kodzik, Andrzej Ciereszko, Bożena Szczepkowska, Agata Malinowska, Mariola Aleksandra Dietrich

**Affiliations:** 1 Department of Gamete and Embryo Biology, Institute of Animal Reproduction and Food Research, Polish Academy of Sciences, Tuwima 10, Olsztyn, 10-748 Poland; 2 Department of Sturgeon Fish Breeding, Inland Fisheries Institute in Olsztyn, Pozezdrze, Pieczarki, 11-610 Poland; 3 Mass Spectrometry Laboratory, Institute of Biochemistry and Biophysics, Polish Academy of Sciences, Pawinskiego 5a, Warsaw, Warszawa, 02-106 Poland

**Keywords:** *Acipenser baerii*, Ovarian fluid, Eggs, Proteome, Mass spectrometry

## Abstract

**Background:**

Sturgeon species are living fossils that exhibit unique reproductive characteristics, and elucidation of the molecular processes governing the formation and quality of sturgeon eggs is crucial. However, comprehensive data on the protein composition of sturgeon ovarian fluid (OF) and eggs and their functional significance are lacking. To address this knowledge gap, the aim of the present study was to conduct a comprehensive comparative proteomic analysis of Siberian sturgeon OF and eggs using liquid chromatography–mass spectrometry (LC–MS/MS).

**Results:**

A total of 617 proteins were identified in OF, and 565 proteins were identified in eggs. A total of 772 proteins showed differential abundance. Among the differentially abundant proteins, 365 were more abundant in OFs, while 407 were more abundant in eggs. We identified 339 proteins unique to OFs and 287 proteins specific to eggs, and further investigated the top 10 most abundant proteins in each. The functional annotation of the OF proteins highlighted their predominant association with immune system processes, including the complement and coagulation cascade, neutrophil and leukocyte-mediated immunity, cholesterol metabolism, and regulation of the actin cytoskeleton. Analysis of egg proteins revealed enrichment in metabolic pathways, such as oxidative phosphorylation and fatty acid metabolism, and protein ubiquitination and translation. OF-specific proteins included extracellular matrix and secretory vesicles, and eggs were enriched in proteins localized to mitochondria and ribosome components.

**Conclusions:**

This study presents the first comprehensive characterization of the protein composition of sturgeon OF and eggs and elucidates their distinct functional roles. These findings advance our understanding of sturgeon reproduction, OF-egg signaling and the origin of OF proteins. The mass spectrometry proteomics data have been deposited in the ProteomeXchange Consortium with the dataset identifier PXD044168 to ensure accessibility for further research.

**Supplementary Information:**

The online version contains supplementary material available at 10.1186/s12864-024-10309-y.

## Background

Sturgeon ancestors existed more than 200 million years ago, and the extant species are living fossils [1] with unique reproductive systems, including gametes, compared to modern teleost fish. For example, sturgeon eggs are encased by a large and thick (50 μm) egg envelope composed of three or four distinct layers compared to the typical one or two envelope layers found in teleost fish. The different structures and numerous micropyles, from 3 to 15, of sturgeon eggs are unusual among fish [2] because the eggs of teleost fish possess only a single funnel-shaped micropyle. Sturgeons are important from an evolutionary perspective and highly valued for their black caviars and high-quality meat. Unfortunately, overfishing for meat and caviar production has led to a severe decline in the sturgeon population, and 27 sturgeon species are currently listed as endangered on the Red List. Therefore, gaining a deeper understanding of the molecular processes underlying sturgeon egg formation and quality is of great scientific and practical importance.

Ovarian fluid (OF) is the maternally derived fluid that surrounds fish eggs, and it plays an important role in creating an optimal environment for egg maturation and fertilization success. OF modulates sperm velocity as a mechanism of cryptic female choice [3]. The biochemical composition of sturgeon OF, including ions, proteins, amino acids and sugars, supports and protects fish gametes against the harmful effects of low freshwater osmolality [4]. The pH of OF and the contents of potassium, sodium and calcium ions are similar to other fish species, but osmolarity varies between fish species. Sturgeon OF has an average osmolality of approximately 200 mOsm kg^− 1^, and salmonids exhibit an average osmolality of greater than 250 mOsm kg^− 1^ [5]. The concentration of proteins in sturgeon OF is greater (2.41 ± 0.30 mg mL^− 1^ for Siberian sturgeon) [6] than that cyprinids (1.58 mg mL^− 1^ for bleak) [7]. There is a gap in our knowledge for the identification of particular proteins of sturgeon OF, which is a prerequisite for a better understanding of their specific roles.

Despite the importance of sturgeon oocytes for aquaculture, little information is available on their protein composition. Proteomic techniques are powerful tools for studying proteins on a large scale and have been successfully used to identify egg proteins in various fish species, such as pikeperch (1296 proteins) [8] and zebrafish (2535 proteins) [9]. Similarly, OF proteins have been identified in chinook salmon (174 proteins) [10], pikeperch (796 proteins) [8] and rainbow trout (54 proteins) [11]. However, only a limited number of proteins have been identified in the OF and eggs of sturgeon species, such as sterlets and Persian sturgeon, and protein counts ranged from 30 to 80 [12, 13]. A comprehensive proteomic study of sturgeon eggs and their environment is lacking because most studies focused solely on the OF or eggs. A comprehensive approach would elucidate the relationship between the OF and eggs. Only one recent proteomic study on pikeperch (a teleost fish) investigated OF and eggs and addressed this issue [8]. In-depth proteomic studies of eggs successfully identified potential biomarkers for egg quality in pikeperch [8] and zebrafish [9]. Comparative proteomic analysis of sturgeon OF and eggs allows determination of the important roles of proteins in the female reproductive system, development and ovarian physiology of fish.

The ecological and economic importance of sturgeon support a clear need to examine the proteomic composition of the OF and eggs of sturgeon species. Therefore, the present study provides a comprehensive overview of the OF and egg proteomes of Siberian sturgeon and compared their proteomes using an efficient LC‒MS/MS approach to elucidate their composition and functional significance. Western blot analysis was used to validate proteins specific to the OF and eggs. This knowledge provides new information on the factors influencing egg quality and reproductive success and may have implications for biomarker discovery and aquaculture practices.

## Results

### Characteristics of sturgeon OF and egg quality

The protein concentration, osmolality and pH of the OF were 2.49 ± 0.28 mg mL^− 1^, 235 ± 11 mOsm kg^− 1^ and 7.74 ± 0.01, respectively. The eggs collected for analysis were characterized by high quality with a fertilization rate at the second cleavage (4 h postfertilization) of 97.5 ± 0.3% and a hatching rate of 78.4 ± 6.5%. The parameters for all five females are provided in Supplementary Table S1.

### LC‒MS/MS identification of OF and egg proteins

In the OF, a total of 732 proteins, containing 3519 peptides, were identified from 12,721 MS/MS matched spectra, and 692 proteins, comprised of 3150 peptides, were identified in eggs from 12,406 MS/MS matched spectra with high confidence (FDR < 1%). Among these identified proteins, 617 and 565 proteins with a minimum of two peptides were detected in at least three of five biological replicates of the OF and eggs, respectively (Supplementary Table S2). The mass spectrometry proteomics data were deposited into ProteomeXchange with the dataset identifier PXD044168. The list of the 10 most abundant proteins (exponentially modified protein abundance index (emPAI) ≥ 9) in the OF and eggs is presented in Table 1.

**Table 1:** The 10 most abundant proteins in the ovarian fluid (OF) and eggs of Siberian sturgeon (*exponentially modified protein abundance index* (emPAI) ≥ 9)

Protein	Gene name	Calculated MW (kDa)	Accession number	Organism	emPAI
**OF**					
serum albumin 2-like	*ALB*	69.1	XP_033869101.2	*Acipenser ruthenus*	420.7
apolipoprotein A-I-like	*APOA1*	30.3	RXM92209.1	*Acipenser ruthenus*	99.0
serotransferrin isoform 3	*TF*	77.1	QHQ72345.1	*Acipenser ruthenus*	65.6
hemoglobin subunit beta-2-like, partial	*HBE1*	16.2	XP_033887121.2	*Acipenser ruthenus*	30.6
hemopexin precursor, partial	*HPX*	50.7	QOI31294.1	*Acipenser gueldenstaedtii*	17.9
nucleoside diphosphate kinase A-like	*NME2*	17.2	XP_033895527.2	*Acipenser ruthenus*	14.9
fish-egg lectin-like isoform X1	*FEL*	26.6	XP_033912133.2	*Acipenser ruthenus*	10.6
alpha-1-antitrypsin homolog	*SERPINA1*	47.5	XP_033892557.2	Acipenser ruthenus	9.1
histone H3-like	*H3C13*	11.4	XP_033853079.1	Acipenser ruthenus	9.0
actin, cytoplasmic 2	*ACTG1*	41.7	XP_033886943.1	*Acipenser ruthenus*	9.0
***EGGS***					
vitellogenin-like (similar to vitellogenin *AB2a [Acipenser schrenckii])*	*vtg2*	193.6	XP_033858533.2	*Acipenser ruthenus*	73.4
nucleoside diphosphate kinase A2 isoform X2	*NME2*	17.2	XP_033895521.1	*Acipenser ruthenus*	38.8
ubiquitin carboxyl-terminal hydrolase isozyme L1	*UCHL1*	24.9	RXM35207.1	*Acipenser ruthenus*	18.3
vitellogenin-like isoform X2 (similar to vitellogenin * AB1 [A. schrenckii])*	*vtg1*	194.3	XP_034780761.1	*Acipenser ruthenus*	14.1
cofilin-2-like	*CFL2*	18.7	RXM93845.1	*Acipenser ruthenus*	14.2
cystatin-B-like	*CSTB*	11.3	XP_033864077.1	*Acipenser ruthenus*	12.9
zona pellucida sperm-binding protein 3-like	*ZP3*	28.9	XP_034773453.1	*Acipenser ruthenus*	10.0
peroxiredoxin-1	*PRDX1*	23.7	XP_033885460.2	*Acipenser ruthenus*	9.0
creatine kinase B-type-like	*CKB*	42.6	XP_033897145.1	*Acipenser ruthenus*	9.0
triosephosphate isomerase B	*TPI1*	26.9	XP_033899162.1	*Acipenser ruthenus*	9.0

### Functional annotation of the OF and egg proteomes

To elucidate the biological functions of the sturgeon OF and egg proteins, we performed a search in the NCBI database and mapped 602 and 553 proteins from the OF and eggs, respectively, to 465 and 471 unique human gene homologs, respectively due to the presence of multiple homologs of the same human protein. Among the identified proteins, gene homologs were not found for 30 and 16 proteins of the OF and eggs, respectively (mostly vitellogenins (VTGs), fish-egg lectin-like, riboflavin-binding protein, protein rapunzel-like, type-4 ice-structuring protein, cell-surface glycoprotein 1, microtubule-associated protein futsch, high choriolytic enzyme 1, gonadal soma-derived growth factor, glycine-rich cell wall structural protein 1.8, stonustoxin and 15 uncharacterized proteins).

**Fig. 1:**
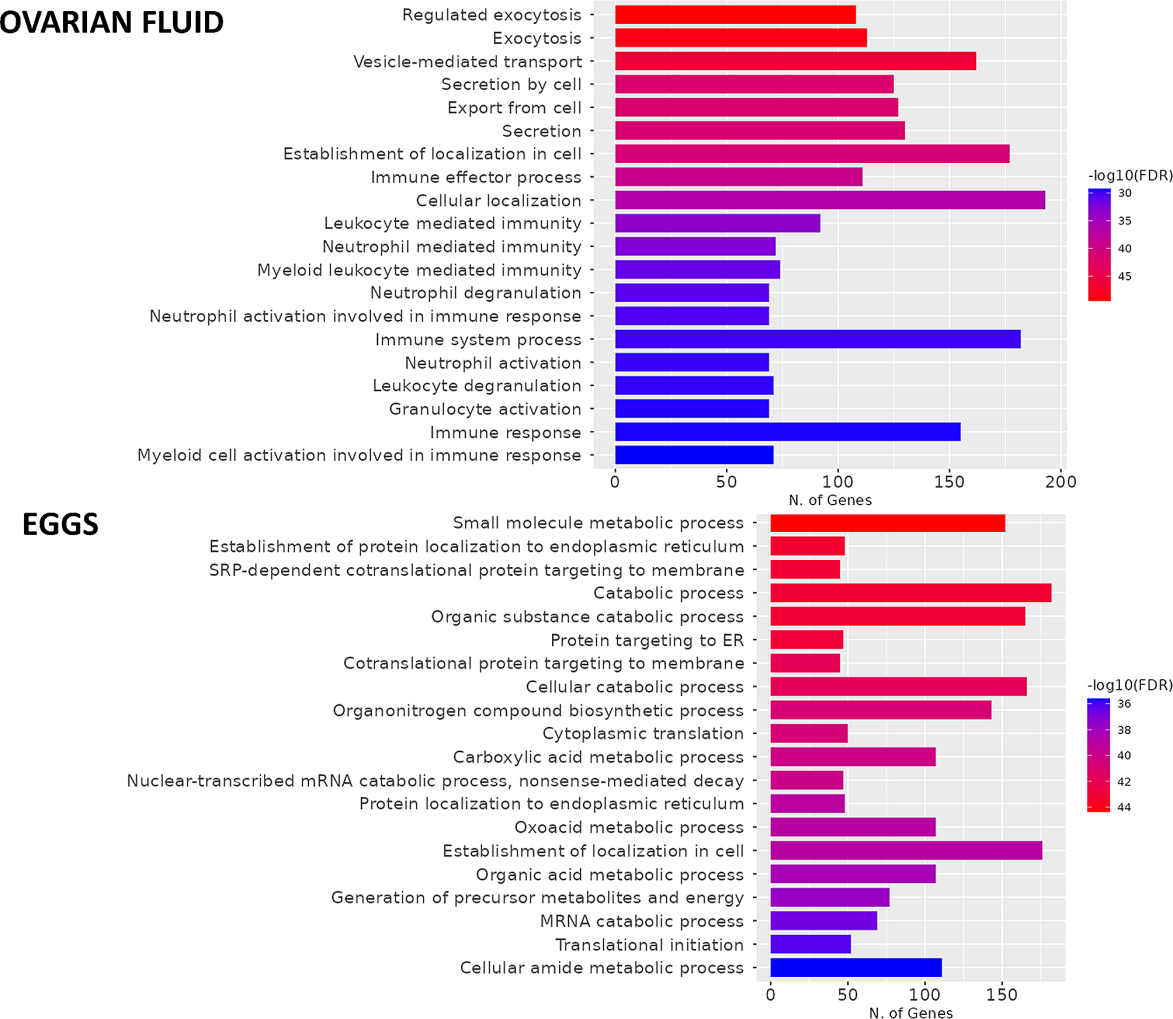
GO biological process enrichment analysis of total identified proteins in the ovarian fluid (OF) and eggs of Siberian sturgeon. Most OF proteins were associated with exocytosis and secretion and immune system processes, but the top-ranked biological processes were cellular metabolic and catabolic processes in eggs

GO analysis of biological process terms revealed that most of the OF proteins were associated with exocytosis and secretion (162 proteins) and immune system processes (182 proteins), especially neutrophil-, leucocyte- and granulocyte-mediated immunity, and various cellular metabolic and catabolic process-related terms (182 proteins) and protein localization, targeting and translation (48 proteins) were enriched among the egg proteins (Fig. 1). Enrichment analysis using Ingenuity Pathway Analysis (IPA) identified 40 and 58 canonical pathways that were significantly enriched in the OF and eggs, respectively. The top 10 canonical pathways are presented in Table 2. KEGG pathway analysis highlighted similar pathways. The full functional annotations for the OF and eggs are available in Supplementary Tables S3 and S4, respectively.

**Table 2:** Top 10 canonical pathways significantly enriched in ovarian fluid (OF) and egg proteins

OF			Eggs		
Pathway	*p* value*	Proteins	Pathway	*p* value*	Proteins
LXR/RXR Activation	1.44E-24	30	EIF2 Signaling	7.32E-44	55
Acute Phase Response Signaling	2.32E-23	34	Oxidative Phosphorylation	1.19E-19	25
FXR/RXR Activation	4.17E-23	29	Protein Ubiquitination Pathway	1.89E-19	29
Complement System	1.49E-21	18	Regulation of eIF4 and p70S6K Signaling	1.89E-14	25
Coagulation System	4.03E-17	15	Inhibition of ARE-Mediated mRNA Degradation Pathway	1.18E-12	22
Actin Cytoskeleton Signaling	1.33E-16	31	Glycolysis I	1.51E-12	11
Clathrin-mediated Endocytosis Signaling	7.43E-15	27	Fatty Acid β-oxidation I	1.64E-12	12
Intrinsic Prothrombin Activation Pathway	1.71E-11	12	Sirtuin Signaling Pathway	6.59E-12	28
Integrin Signaling	3.24E-11	23	TCA cycle II	2.62E-11	9
Epithelial Adherens Junction Signaling	3.24E-11	20	Gluconeogenesis	2.82E-11	10

* The *p*-value associated with a function or a pathway is a measure of the likelihood that the association between a set of focus genes in the experiment and a given process or pathway is due to random chance. The smaller the *p*-value the less likely that the association is random and the more significant the association. In general, a *p*-value (calculated using the right-tailed Fisher exact test) *<* 0.05 indicates a statistically significant, nonrandom association

### Comparison of OF and egg proteins

**Fig. 2:**
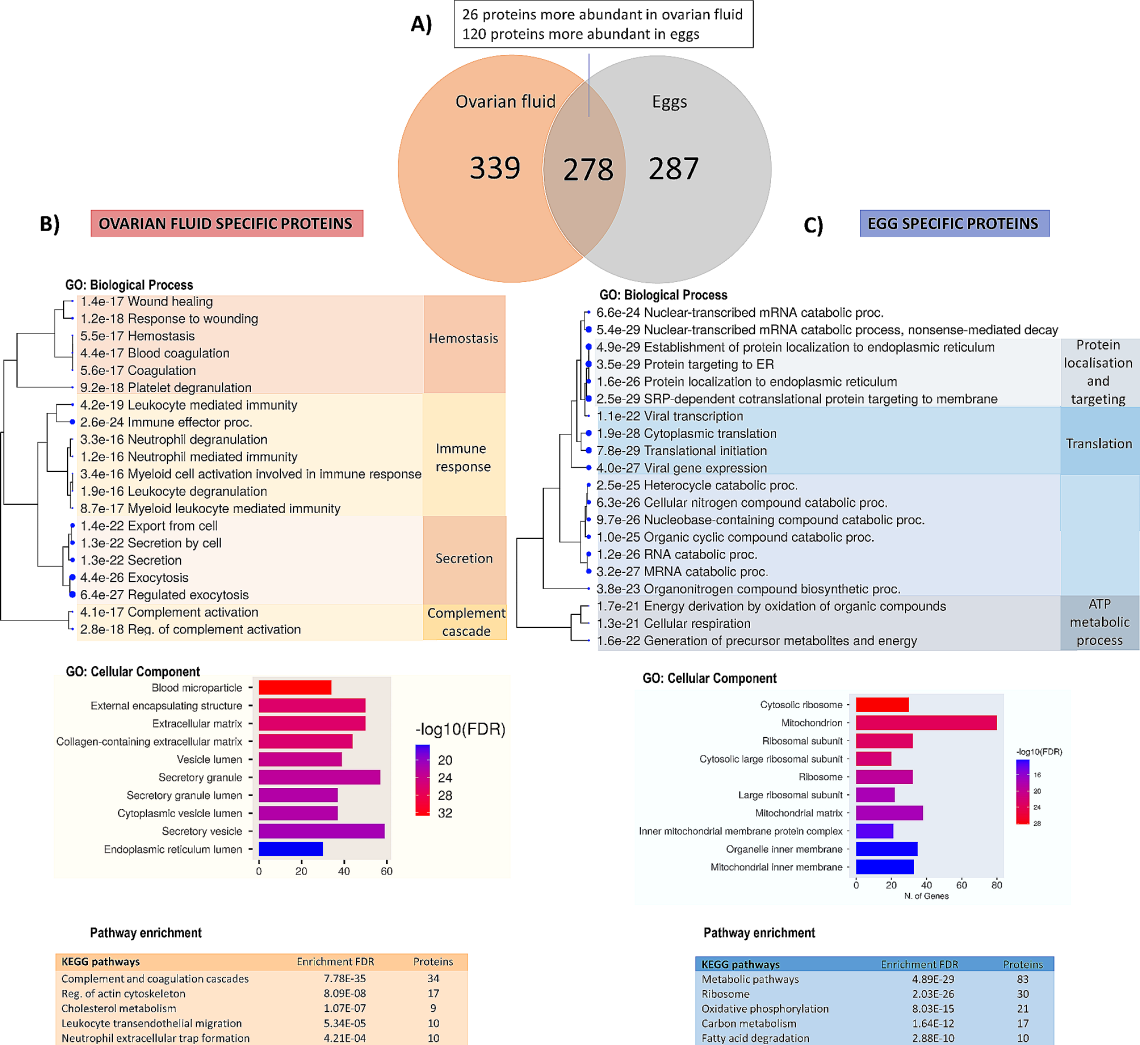
Functional analysis of proteins unique to ovarian fluid (OF) and eggs. Venn diagram representing the overlap of all identified proteins in the OF and eggs of Siberian sturgeon (**A**). GO biological processes and cellular components; KEGG pathway analysis of the OF (**B**) and egg (**C**) proteins. Pathways with many shared genes are clustered together. Larger dots indicate more significant *P* values

Using the emPAI-based estimates of the quantitative relationships within each type of sample, a total of 772 differentially abundant proteins (DAPs) between the OF and eggs were identified, including 365 proteins that were more abundant in the OF and 407 proteins that were more abundant in eggs (Supplementary Table S5). Of these DAPs, 339 proteins were exclusively detected in OF samples, and 287 were unique to eggs (i.e., not detected in the OF samples) (Fig. 2A; Supplementary Tables S6). Among the DAPs shared by the OF and eggs, the top proteins (FC > 20) in OF were albumin (ALB) (140-fold), serotransferrin (TF) (72-fold), hemopexin (HPX) (51-fold), alpha-1-antitrypsin homolog (SERPINA1) (30-fold), and cytosolic nonspecific dipeptidase (CNDP2) (29-fold), where as zona pellucida sperm-binding proteins (ZPs) (40-fold), peroxiredoxin 4 (PRDX4) (31-fold), vitellogenin-like (VTG2) (23-fold), ubiquitin carboxyl-terminal hydrolase isozyme L1 (UCHL1) (23-fold), and endoplasmin (HSP90B1) (20-fold) were highly abundant in the eggs (Supplementary Table S7).

### GO and KEGG enrichment analyses of proteins specific to the OF and eggs

GO term enrichment analysis of the proteins unique to the OF and eggs revealed significant enrichment of 500 and 338 biological processes, respectively (Supplementary Table S8 and Table S9). We present the top 20 significantly enriched GO biological processes, which shows that most proteins unique to the OF were associated with exocytosis (61 proteins) and immune and stress response (107 proteins), especially leukocyte- and neutrophil-mediated immunity, complement activation and coagulation (Fig. 2B, C). In contrast, metabolic processes (100 proteins; including catabolic processes, such as RNA catabolic process and nitrogen compound catabolic process and energy derivation), protein localization and targeting (31 proteins), and translation (33 proteins) were enriched in eggs. KEGG pathway analysis revealed that proteins involved in complement and coagulation cascades (34 proteins), regulation of the actin cytoskeleton (17 proteins), cholesterol metabolism (9 proteins) and immune-related pathways were specifically enriched in the OF, and metabolic pathways (83 proteins), including oxidative phosphorylation (21 proteins), carbon metabolism (17 proteins), fatty acid degradation (10 proteins) and ribosomes (30 proteins), were most enriched in eggs (Fig. 2B, C). Among the proteins involved in the complement cascade, we identified proteins related to the classical, alternative and lectin pathways (Supplementary Figure S1). IPA highlighted similar pathways and emphasized the representation of proteins involved in eukaryotic initiation factor (EIF) signaling and protein ubiquitination pathways for egg-specific proteins. IPA revealed functional enrichment of OF proteins involved in organismal survival, inflammatory response, immune cell trafficking, hematological system development and organismal development, and egg-specific proteins were mostly involved in tissue morphology, embryonic development, organismal survival, connective tissue development and nervous system development (Table 3).

**Table 3:** Functional analysis of proteins specific to ovarian fluid (OF) and eggs using Ingenuity pathway analysis (IPA)

OF specific proteins			Egg specific proteins		
**Top canonical pathway**	***p*** value*	**Proteins**	**Top canonical pathway**	***p*** value*	**Proteins**
Complement System	3.73E-26	18	EIF2 Signaling	4.17E-31	36
Acute Phase Response Signaling	3.34E-25	29	Oxidative Phosphorylation	7.28E-19	20
LXR/RXR Activation	5.02E-22	23	Protein Ubiquitination Pathway	8.14E-12	14
FXR/RXR Activation	8.98E-22	23	Fatty accid β-oxidation	1.14E-12	10
Coagulation System	4.37E-19	14	Regulation of eIF4 and p70S6K Signaling	1.14E-12	16
**Top molecular and cellular functions**	p value*	**Proteins**	**Top molecular and cellular functions**	p value*	**Proteins**
Organismal Survival	2.5E-28-1.3E-16	135	Tissue Morphology	3.4E-10-9.7E-03	25
Inflammatory Response	6.7E-28-4.5E-09	148	Embryonic Development	1.2E-06-9.2E-04	9
Immune Cell Trafficking	1.4E-26-3.3E-09	87	Organismal Survival	4.8E-05-4.8E-05	75
Hematological System Development and Function	2.1E-25-3.3E-09	119	Connective Tissue Development and Function	2.7E-04-6.2E-03	9
Organismal Development	1.2E-23-3.9E-09	129	Nervous System Development and Function	4.6E-04-9.7E-03	20

* the *p* value associated with a function or a pathway is a measure of the likelihood that the association between a set of focus genes in the experiment and a given process or pathway is due to random chance. The smaller the *p* value, the less likely that the association is random and the more significant the association. In general, a *p* value (calculated using the right-tailed Fisher exact test) < 0.05 indicates a statistically significant, nonrandom association

**Fig. 3:**
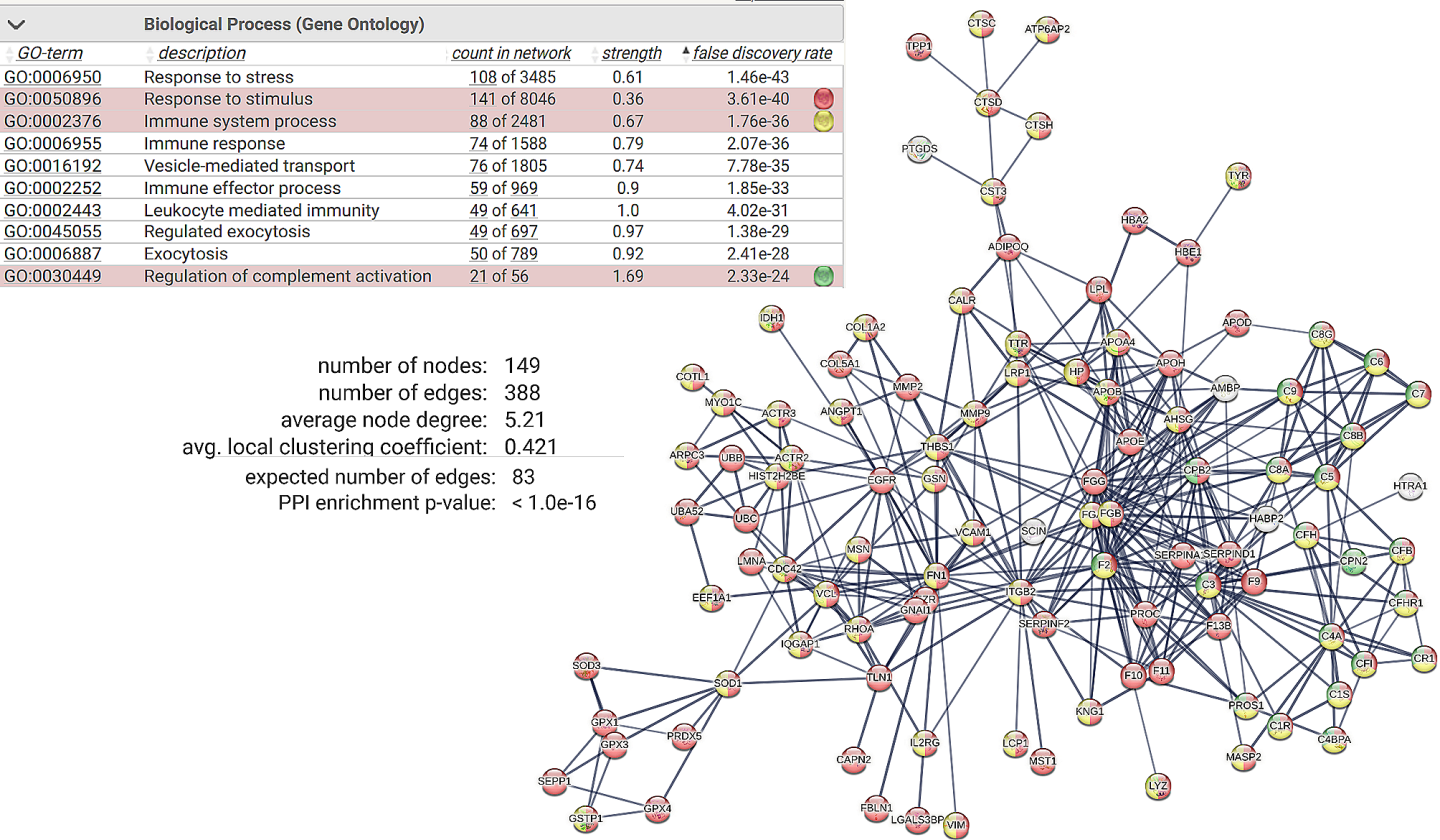
Results of the Search Tool for the Retrieval of Interacting Genes/Proteins (v 11.0) analysis showing the protein association network of proteins specific to ovarian fluid assigned to immune system processes with a high confidence score. The nodes correspond to the proteins, and the edges represent the interactions (thick lines indicate a high score > 0.9; thin lines indicate a medium score > 0.7). Model statistics are presented on the left. An explanation of the edge colors is provided above the figure

**Fig. 4:**
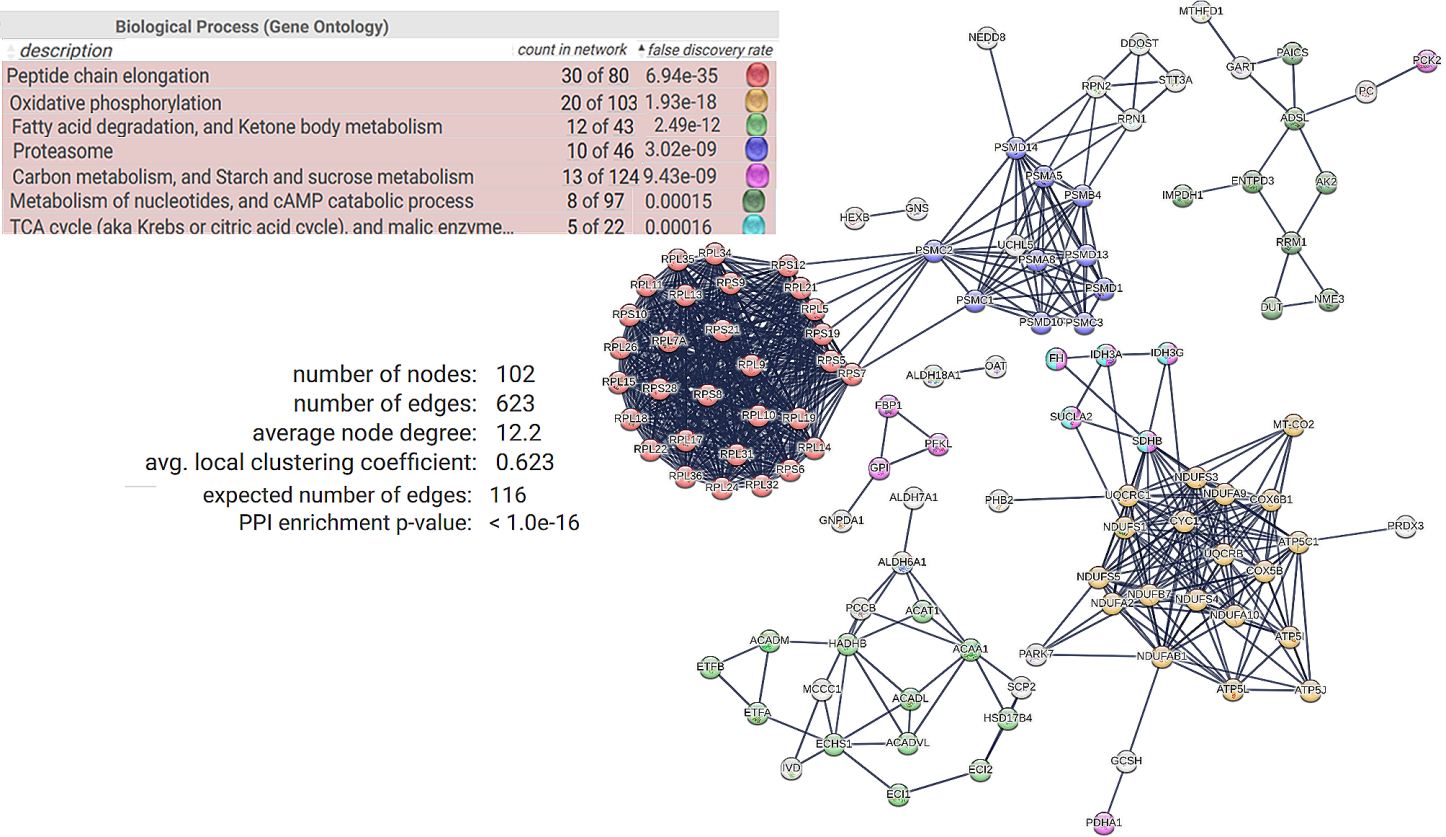
Results of the Search Tool for the Retrieval of Interacting Genes/Proteins (v 11.0) analysis showing the protein association network of proteins specific to eggs assigned to metabolic processes with a high confidence score. The nodes correspond to the proteins, and the edges represent the interactions (thick lines indicate a high score > 0.9; thin lines indicate a medium score > 0.7). Model statistics are presented on the left. An explanation of the edge colors is provided above the figure

Among the OF-specific proteins related to immune and stress responses, there was a high degree of connectivity between each other (Fig. 3). Among the egg-specific proteins linked to metabolism, six distinct interaction networks were found, among which 28 ribosomal proteins and proteins involved in oxidative phosphorylation (19 proteins) exhibited the highest degree of connectivity (Fig. 4).

### OF and egg proteins associated with reproduction

ShinyGo and GO analyses revealed that 102 OF proteins and 117 egg proteins were involved in reproductive processes. An additional search of OF and egg proteins using the Panther database (http://www.pantherdb.org/) revealed their involvement in the ovulation cycle, oocyte maturation, prevention of polyspermy, ovarian aging, fertilization, sperm-egg interaction and embryonic development. All identified proteins and their female reproductive functions are available in Supplementary Table S10.

### Validation of mass spectrometry data using Western blotting

**Fig. 5:**
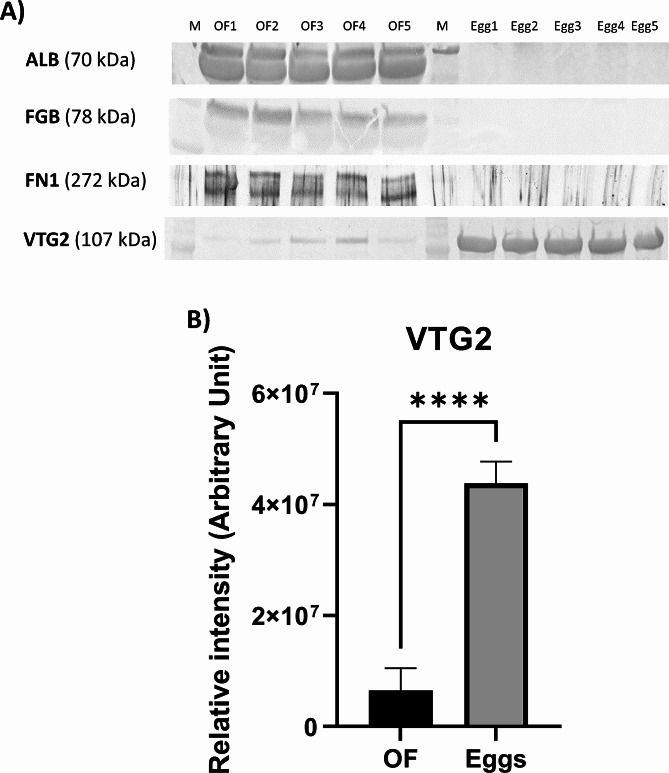
Validation of proteins specific to ovarian fluid (OF) and eggs, such as albumin (ALB), fibrinogen (FGB), fibronectin-1 (FN1), and vitellogenin 2 (VTG2), using Western blot analysis (**A**). The full-length blots are presented in Supplementary Fig. S2. A quantitative comparison of VTG2 in the OF and eggs (**B**). The data are presented as the means ± S.D. (*n* = 5 in OF and eggs). Statistical analysis was performed using Student’s t test; ****p ≤ 0.0001. The intensity of the protein bands on the TGX Stain-Free gels was analyzed using Image Lab 6 software (Bio-Rad)

To validate the LC‒MS/MS results, we selected four proteins for further analysis using 1D Western blotting: ALB, fibrinogen beta chain (FGB), fibronectin 1 (FN1) and vitellogenin-like (XP_033858533.2), which shows 97.89% identity to vitellogenin AB2a from *Acipenser schrenckii*, corresponding to the fish VTG2 [14] (Supplementary Figure S2). As presented in Fig. 5, the changes in the abundance of the selected proteins were consistent with the changes in the LC‒MS/MS analysis. Western blot analysis confirmed the absence of FGB and FN1 proteins in the eggs and demonstrated their exclusive presence in the OF (Fig. 5A). A strong signal of ALB was also detected in OF. However, due to its low concentration in eggs (140 times lower than in the OF), it was not detected using the current conditions (i.e., below the limit of detection). Western blot analysis revealed a six-fold greater abundance of VTG2 in eggs compared to the OF (Fig. 5B). The full-length blots are presented in Supplementary Figure S2. The specificity of the antibodies used was confirmed using mass spectrometry analysis (Supplementary Table S11).

## Discussion

The present study presents the first comprehensive characterization of the OF and mature egg proteomes of sturgeon and their comparison. Our findings contribute to the largest proteomic catalog of the OF and eggs in sturgeons (*Actinopterygii*), with a total of 602 and 553 proteins identified in the OF and eggs, respectively. Pathways related to mRNA translation (EIF signaling), protein degradation (ubiquitin‒proteasome pathway) and metabolic pathways (oxidative phosphorylation, glycolysis, fatty acid β-oxidation, and the sirtuin signaling pathway) were the most significant canonical pathways in eggs. OF was enriched in various pathways, such as LHR/RXR activation, acute phase response signaling, complement and coagulation system, actin cytoskeleton signaling, clathrin-mediated endocytosis signaling, and integrin and epithelial adherens junction signaling. We also identified proteins that were unique to the OF and eggs, which elucidated their distinct molecular profiles.

### Most abundant proteins in the OF and eggs and their functions

The most abundant proteins in the sturgeon OF were predominantly involved in the humoral innate immune response, including acute phase proteins (APPs), antimicrobial peptides (AMPs) and pattern recognition receptors (PRRs). Acute phase response proteins (ALB, TF, HPX, SERPINA1 and apolipoprotein A-I (APOA1)) are multifunctional proteins that participate in various processes, such as iron ion homeostasis (TF, ALB, HPX), oxidative protection (TF, HPX, ALB, APOA1), lipid metabolism (APOA1) and protease activity regulation (SERPINA1). Histone H3 (H3) and hemoglobin subunit beta (HBB) are precursors of AMPs, which play a fundamental role in innate immunity by killing pathogens and modulating the immune response in fish [15]. Fish egg lectin (FEL) was previously identified in the eggs of several teleost fish species, and it acts as a PRR that specifically binds to bacteria and as an opsonin to protect developing embryos/larvae from pathogenic invasion [16]. Among the abundant proteins in sturgeon OF, actin cytoplasmic 2 (ACTG1) and nucleoside diphosphate kinase A-like (NME1), which play important roles in oogenesis and embryo development in fish, were identified [17, 18]. The functions of ACTG1 and NME1 may also be linked to the immune response because ACTG1 is involved in the motility, phagocytosis and antigen presentation of immune cells [19], and NME1 is involved in T-cell activation [20]. Most of the dominant proteins in sturgeon OF (ALB, TF, HPX, HBB, SERPINA1, APOA1) were also found among the highly abundant proteins in the OF of teleost fish [8, 10, 11], which suggests that the general protective mechanisms of oocytes against pathogens and oxidative stress are evolutionarily conserved in the OF and similar to teleost fish. However, unlike teleost fish, the levels of histones H1 (H1), ACTG1, NME1 and FEL were more abundant in sturgeon OF, which indicates the importance of these proteins in the development and protection of sturgeon oocytes and embryos.

The dominant egg proteins were associated with various functions, including development, immune response, antioxidative protection, metabolism and fertilization. Similar to teleost fish, numerous VTG family members were among the most abundant proteins in sturgeon eggs [3, 8, 10–13]. VTGs play multiple roles in providing an energy reserve for developing embryos and protecting them against microbial attacks and oxidative stress [21]. Cystatin-B (CSTB) is a reversible inhibitor of cysteine proteases, and it is involved in cellular protection against proteolysis and immune defense mechanisms in fish eggs, which indicates internal and external protection of eggs against pathogens. The abundance of PRDX1, which is an enzyme that reduces hydrogen peroxide, highlights the importance of the peroxiredoxin system in protecting sturgeon eggs against oxidative stress. ZP3 is an important component of the white sturgeon egg envelope, and it plays a role in fertilization [12, 22]. ZP3 functions in teleosts are also related to oogenesis and embryonic development [23–25]. Cofilin-2 (CFL2) is an actin-depolymerizing factor that is essential for actin cytoskeleton organization in fish oocytes and eggs. CFL2 may function with ZPs in envelope hardening and egg adhesion after fertilization in sturgeon [26]. NME was also identified as a major protein in the OF in this study, and UCHL1 is a deubiquitinating enzyme in the ubiquitin‒proteasome system that is involved in oocyte and embryo development in fish. Ubiquitin-related genes are used as biomarkers of good-quality sea bass eggs [17, 27]. Among the abundant proteins, we also identified the metabolic enzymes creatine kinase B (CKB) and triosephosphate isomerase (TPI), which are involved in energy production via the creatine-phosphocreatine system and glycolysis, respectively. Notably, our study revealed differences in the composition of major proteins in eggs between teleosts [8] and sturgeons (current study). Only VTGs and ZPs were common, which may reflect the distinct structure of sturgeon oocytes, including the presence of multiple micropyles, a complex envelope structure, and a specific embryo development pattern (holoblastic cleavage) that is more similar to Xenopus than teleost fish.

### Role of proteins unique to the OF in the immune response

Bioinformatic analysis revealed that most proteins unique to the OF were associated with immune system processes, including humoral and cellular responses. Our findings indicated the presence of three activation pathways of the complement system in sturgeon OF: classical (C1r-C1s, C1q, C2, and C4B); lectin (mannan-binding lectin serine protease 2 (MASP2) and C4B); and alternative (complement factor H-related protein 1 (CFHR1) and complement factor B (CFB)). We also identified component C3, which is crucial for these pathways, and components of the common terminal pathway (C5, C6, C7, C9, C8A, C8B, anC8G) and regulatory proteins (complement receptor type 1 and 2 (CR1, CR2), complement factor I (CFI), complement factor H (CFH), C4b-binding protein alpha chain (C4BP)), which tightly control complement system activity under normal and pathological conditions. The complement system interacts with the coagulation system identified in our study and serves as the “first line of defense” during infection to initiate opsonization, regulate adaptive immunity, enhance circulating immunoglobulins, promote immune cell recruitment, and facilitate direct cell membrane lysis [28]. We also identified positive (haptoglobin (HP), ceruloplasmin (CP), alpha-2-antiplasmin (SERPINF2), SERPINA1, heparin cofactor 2 (SERPIND1), fibrinogen alpha chain (FGA), fibrinogen gamma chain (FGG), FGB, and plasminogen (PLG)) and negative (interalpha-trypsin inhibitor heavy chain H2 and H3 (ITIH2, ITIH3), APOA1, alpha-2-HS-glycoprotein (AHSG), and histidine-rich glycoprotein (HRG)) APPs that are involved in defense-related mechanisms, including tissue repair, protection against ROS, metal chelation, activation of the complement system, enzyme neutralization and pathogen killing [29]. Proteins related to the coagulation cascade and acute inflammatory response in the OF may play a role in ovulation, which is considered a controlled inflammatory reaction in mammals and fish [30, 31]. Other humoral components identified in our study included lysozyme and cathelicidin, which are known antimicrobial peptides [32]. PRRs are part of the immune system of sturgeon OF. Our findings also suggest that proteins in sturgeon OF are involved in the activation of macrophages, neutrophil degradation and extracellular trap formation. These findings indicate that neutrophils, which are classified as heterophils in sturgeon [33], and macrophages are crucial elements of the cellular innate immune system of the OF. In summary, the complex immune system in sturgeon OF, which is composed of a variety of humoral and cellular components, strongly protects gametes against pathogens, scavenges free radicals, and may play important roles in ovulation and maintaining homeostasis following inflammation.

### OF proteins involved in lipid metabolism

Our study identified several proteins involved in lipid metabolism in the OF, including lipoproteins (APOA1, apolipoprotein B-100 (APOB), apolipoprotein C-I (APOC1), apolipoprotein D (APOD), beta-2-glycoprotein 1 (APOH), apolipoprotein E (APOE)), lipoprotein and phospholipase receptors, phospholipase inhibitors and lipases. Lipoproteins, such as very low- and high-density lipoprotein (VLDL and HDL, respectively), are present in fish blood and the OF play a crucial role in lipid metabolism and transport, specifically cholesterol and triglycerides, to the ovary, where they are taken up by ovarian somatic cells and oocytes for energy production and processes related to oocyte maturation and development [34–36]. Our results suggest that the uptake of lipoproteins and VTG2 by eggs is facilitated by low-density lipoprotein receptor-related proteins (LRP1, LRP2, LRP6) and the very low-density lipoprotein receptor (VLDLR), which is similar to other fish species [37]. Although these receptors are typically found on the cell surface to mediate lipoprotein internalization, fragments or shed forms of these receptors may be detected in the OF. The presence of these receptors in the OF was detected in our study and other fish species [3, 8]. Our analysis also revealed the presence of lipoprotein lipase (LPL) in the OF, which is a key enzyme for hydrolyzing triglycerides from VLDL and LDL and generates essential free fatty acids and glycerol for lipid uptake and energy production in fish oocytes [38, 39]. The identification of a phospholipase A inhibitor suggests the presence of a regulatory mechanism that controls the activity of phospholipase A, which is responsible for the hydrolysis of phospholipids. The secretory phospholipase A2 receptor (PLA2R1) may further contribute to lipid metabolism by facilitating the binding and internalization of phospholipase A2 enzymes. Overall, our results highlight the tight regulation of lipid transport and metabolism in sturgeon OF, which involves a specialized set of proteins. These proteins play a significant role in lipid incorporation and accumulation, which may be important for prolonged oocyte maturation and development in sturgeon.

### Extracellular matrix (ECM) proteins in the OF

ECM proteins play a crucial role by providing structural support, facilitating cell-matrix interactions, and regulating various processes involved in follicular development and oocyte maturation. The identification of specific ECM proteins in the OF, such as collagen alpha-1 (XI) chain (COL11A1), fibulin-1 (FBLN1), FN1, collagen alpha-1(V) chain (COL5A1), collagen alpha-3(VI) chain (COL6A3), lumican (LUM), prelamin-A/C (LMNA), proteoglycan 4 (PRG4), and chondroitin sulfate proteoglycan 4 (CSPG4), highlights their essential role in maintaining the integrity of ovarian tissue, particularly the basal lamina and theca. These structural proteins form an ECM framework that supports the growth and development of ovarian follicles in sturgeon, which is vital for housing oocytes [40]. In addition to structural proteins, other ECM components, such as cathepsins (pro-cathepsin H (CTSH), dipeptidyl peptidase 1 (CTSC), and cathepsin D (CTSD)), PLG, matrix metalloproteinase-2 and 9 (MMP2, MMP9), and their metalloproteinase inhibitor 4 (TIMP4), are involved in follicular remodeling and the breakdown of the follicle wall during ovulation [41–43]. Recent studies also suggested the involvement of the plat/plasmin system in ECM remodeling during ovulation in sturgeon [44], which further supports the dynamic nature of the ECM in the sturgeon ovary. ECM proteins, such as myocilin (MYOC), thrombospondin-4 (THBS4), cadherin 13 (CDH13), leucine-rich repeat-containing protein 15 (LRRC15), and tetranectin (CLEC3B), may contribute to cell adhesion and signaling within the ovary in sturgeon. They facilitate important cell‒cell and cell‒matrix interactions that regulate the signaling pathways necessary for oocyte maturation, ovulation, and tissue homeostasis [45]. We identified several ECM proteins, including scinderin (SCIN), vinculin (VCL), transforming protein RhoA (RHOA), ezrin (EZR), actin-related protein 2/3 complex subunit 3 (ARPC3), actin-related protein 2 and 3 (ACTR2, ACTR3), ras GTPase-activating-like protein IQGAP1 (IQGAP1), moesin (MSN), gelsolin (GSN), integrin beta-2 (ITGB2), cofilin-1 (CFL1), alpha-actinin-4 (ACTN4), and prothrombin (F2), which are involved in the regulation of the actin cytoskeleton. By modulating the actin cytoskeleton, these ECM proteins may contribute to various processes, including tissue remodeling and the successful release of mature oocytes [46]. Overall, the presence of these ECM proteins in sturgeon OF highlights their indispensable role in multiple processes within the ovary, including follicular structure and development, oocyte maturation, tissue remodeling, and the successful release of mature oocytes.

### Egg proteins associated with transcription and translation processes

Translation and translation processes, specifically EIFs and ribosomal components, are highly important during oogenesis and early embryo development in metazoans [47]. Among the identified proteins, EIF2 signaling and the regulation of EIF4 and p70S6K signaling were the most significant canonical pathways in the sturgeon egg proteome. Notably, we identified the eukaryotic initiation factor 4 A-I and II (EIF4A1 and EIF4A2) proteins, which are responsible for unwinding mRNA secondary structures, facilitating ribosome binding, and initiating translation. We also detected eukaryotic translation initiation factor 4 gamma 3 (EIF4G3), which is an interacting scaffold protein with EIF4A and other initiation factors, and eukaryotic translation initiation factor 5 (EIF5), which facilitates the joining of ribosomal subunits during translation initiation. Other translation-associated proteins were detected, including 30 ribosomal proteins that actively contribute to the structure and function of the ribosome, polyadenylate-binding protein 1 (PABPC1), a poly(A)-binding protein, and phosphatidylinositol 4-phosphate 3-kinase C2A (PIK3C2A), which is a phosphoinositide kinase involved in intracellular signaling pathways, including the regulation of translation. These results further highlight the importance of these proteins in oocyte physiology in sturgeon. Notably, the presence of EIFs has been observed in the eggs of other fish species [8, 9]. Previous studies identified eukaryotic translation initiation factor 3 subunit D (EIF3D) and two ribosomal proteins (large ribosomal subunit protein eL36- RPL36-001, RPL36-002) as markers of high-quality eggs, and an upregulated form of ribosomal protein L22 (RPL22) was associated with poor-quality zebrafish eggs [9]. In summary, our findings highlight the significance of translation and transcription processes in the synthesis of maternal RNAs and proteins crucial for embryo development, oocyte maturation, and fertilization in sturgeon. Because the initial stages of embryo development are under maternal control, our results significantly contribute to this knowledge by revealing several proteins and their involvement in this process.

### Egg metabolism-related proteins

**Fig. 6:**
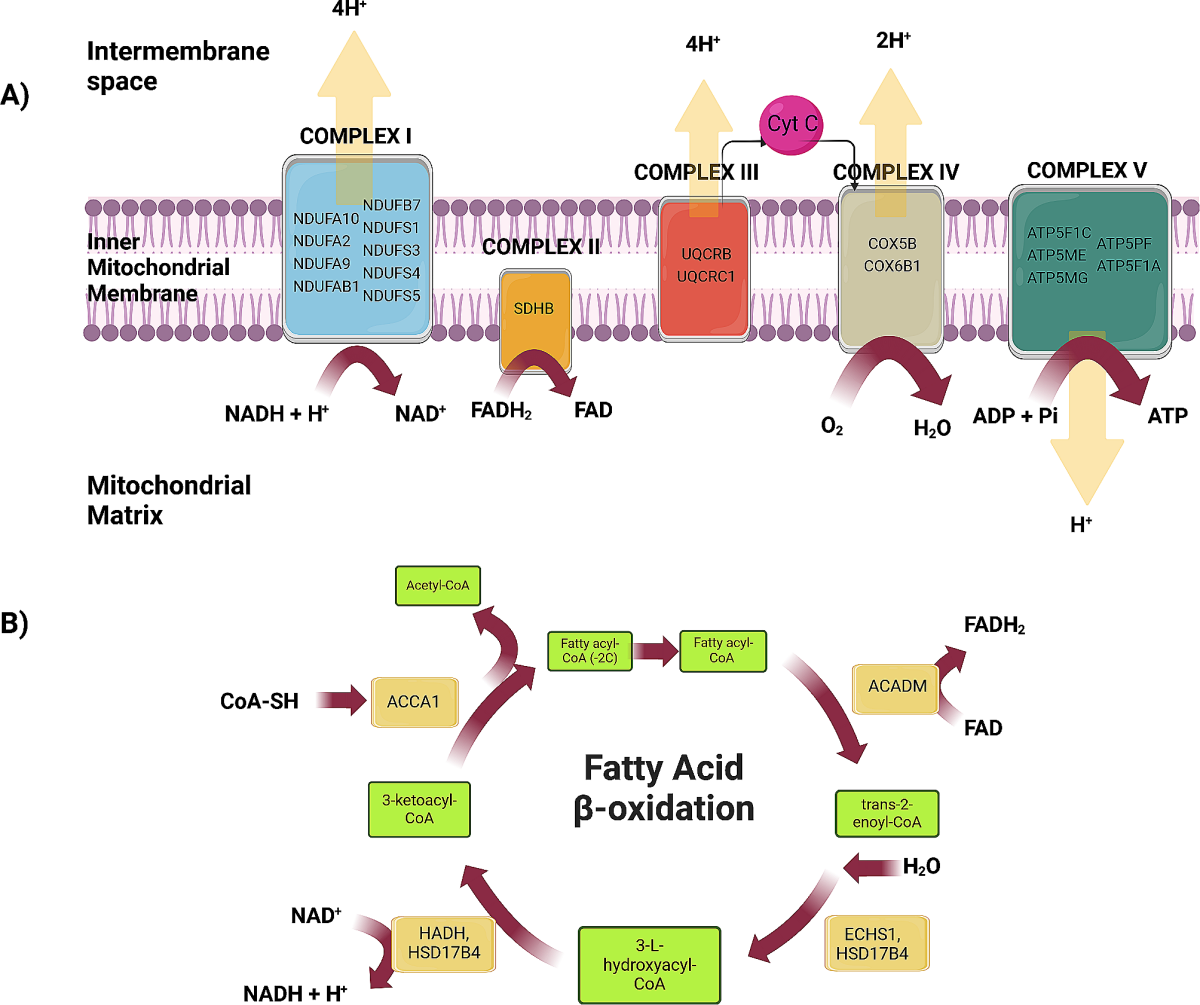
Proteins identified in Siberian sturgeon eggs that participate in mitochondrial oxidative phosphorylation complexes I–V (**A**) and fatty acid $\boldmath{\beta }$-oxidation I (**B**). The scheme was prepared using BioRender software. Descriptions of the gene/protein symbols are presented in Supplementary Table S2

Our study revealed the significance of the oxidative phosphorylation (OxPhos) and fatty acid β-oxidation pathways in sturgeon eggs, which are primarily represented by egg-specific proteins. OxPhos is the final biochemical pathway involved in ATP production within the mitochondria, and we identified proteins involved in the five multiprotein complexes I-V (CI-CV) of OxPhos, including several forms of NAD dehydrogenase (CI), succinate dehydrogenase (CII), cytochromes (CIII, CIV) and ATP synthase (CV) (Fig. 6A). Previous studies highlighted the impaired oxidative phosphorylation in low-quality eggs of rainbow trout, which highlights its importance in fish oocyte maturation [48]. Oxidative metabolism, including OxPhos, has been observed in unfertilized steelhead eggs and during early developmental stages [49]. We identified specific proteins in sturgeon eggs associated with the mitochondrial fatty acid β-oxidation I pathway, which is responsible for the degradation of fatty acids to produce energy [50]. These proteins participate in consecutive reactions, including dehydrogenation (catalyzed by medium-chain specific acyl-CoA dehydrogenase, mitochondrial (ACADM), hydroxylation (enoyl-CoA hydratase, mitochondrial (ECHS1), peroxisomal multifunctional enzyme type 2-HSD17B4), a second dehydrogenation (trifunctional enzyme subunit beta, mitochondrial (HADHB) and HSD17B4)) and thiolysis (3-ketoacyl-CoA-thiolase-ACAA1) (Fig. 6B). We identified other proteins involved in mitochondrial beta-oxidation, such as enoyl-CoA delta isomerase 1 and 2, mitochondrial (ECI1, ECI2), isovaleryl-CoA dehydrogenase, mitochondrial (IVD) and synaptonemal complex protein 2 (SCP2). Mitochondria in zebrafish embryos are active and utilize free fatty acids as substrates for oxidative phosphorylation to provide ATP [51]. Our results suggest that mitochondrial oxidative phosphorylation, in conjunction with fatty acid β-oxidation, plays a crucial role in oocyte maturation, metabolism, and energy homeostasis in chondrostean fish.

### Proteins involved in reproduction

We identified proteins involved in key reproduction processes in sturgeon OF and eggs, such as ovulation, oocyte maturation, prevention of polyspermy, fertilization, and sperm-egg activation. The eggs showed enrichment of common proteins, such as components of the ubiquitination cascade (UCHL1, UCHL3) and heat shock proteins (HSP5, HSP9 and HSP90AB1), which suggests their role in sturgeon oocyte development and regulation [52, 53]. ZP proteins (ZP1-4) were also increased in eggs, and these proteins are vital for oocyte growth, species-restricted fertilization, and preventing polyspermy [54]. Our investigation revealed 53 specific proteins in eggs that were primarily associated with early development processes. Among these proteins, we identified eight components of the 20 S and 26 S proteasomes that are essential for the ubiquitin-dependent protein degradation pathway and oocyte maturation in fish [55] and an oocyte-specific linker histone, protein B4, which is restricted to early development in Xenopus [56]. We also detected proteins, such as sperm acrosome associated 4 (SPACA4), zona pellucida sperm-binding protein 3 receptor (ZP3R) and glucosamine-6-phosphate isomerase 1 (GNPDA1), which are crucial for sperm-egg plasma membrane adhesion, fertilization, and early embryo development [57, 58]. In summary, our comprehensive analysis of sturgeon OF and egg protein profiles provides vital insights into the intricate mechanisms of female reproduction.

### Comparison of OF and seminal plasma proteins

The comparison of Siberian sturgeon OF and seminal plasma (SP) proteins revealed similarities and specificities in their composition (Supplementary Figure S3, Supplementary Table S13). The identification of shared proteins in the OF and SP indicates the convergence of functional roles, particularly in the humoral response, metabolic processes, and cellular detoxification, which are sustained by common major proteins, such as ALB, TF, HPX, APOA1, and FEL [59], which are primarily associated with immune and stress responses. This finding reinforces the significance of the fluid milieu surrounding eggs and spermatozoa in providing a protective and supportive environment for their viability and functionality [8, 10, 11, 60]. The presence of OF-specific proteins involved in immune response processes, cell adhesion and metabolism reinforces its role in protection and oocyte maturation and highlights its potential nutritive contributions to gametes via the VTGs (see above). Conversely, proteins specific to seminal plasma, such as proteins related to developmental processes, metabolism, proteolysis, cell adhesion, and extracellular matrix organization, may be associated with spermatogenesis and sperm maturation. Significantly, our analysis identified specific proteins crucial for fertilization. We identified sperm-egg recognition proteins (ZP1, ZP2, ZP3, ZP4, T-complex protein 1 subunit epsilon (CCT5), folate receptor beta and gamma (FOLR2, FOLR3), voltage-dependent anion-selective channel protein 2 (VDAC2), and astacin-like metalloendopeptidase (ASTL)) in the OF. We identified proteins associated with the binding of sperm to the zona pellucida (acrosin (ACR), cAMP-dependent protein kinase type II-alpha regulatory subunit (PRKAR2A), zona pellucida-binding protein 2 (ZPBP2), T-complex protein 1 subunit gamma and eta (CCT3, CCT7), zonadhesin (ZAN), and arylsulfatase A (ARSA)) in seminal plasma. This finding highlights the distinct and complementary functions of the OF and SP proteins in facilitating successful sperm–egg interactions. The delineation of specific functions attributed to the OF and SP proteins provides valuable insights into their unique contributions to reproductive processes, which enhances our understanding of the complex interplay between these fluids and their impact on gamete function to ultimately influence fertilization success.

## Conclusions

**Fig. 7:**
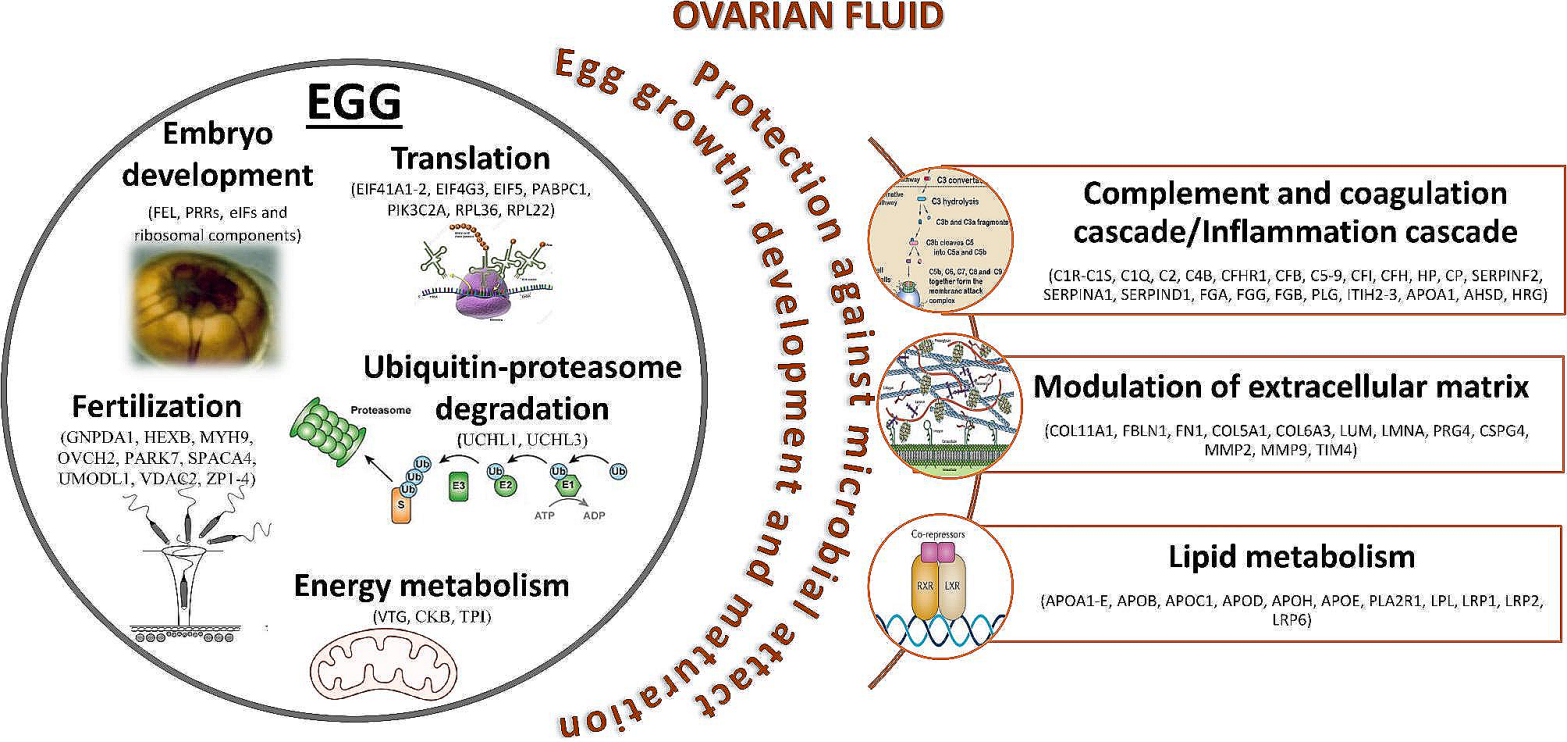
Scheme presenting an overall summary of the unique roles of the identified proteins in the ovarian fluid (OF) and eggs of Siberian sturgeon. OF proteins were associated with the inflammatory cascade, modulation of the extracellular matrix and lipid metabolism, and proteins in the eggs were primarily involved in embryo development, fertilization, translation, ubiquitin - proteasome degradation and energy metabolism. Descriptions of the gene/protein symbols are presented in Supplementary Table S2

Our study presents a comprehensive characterization of the proteomic profiles of the OF and eggs from Siberian sturgeon and provides novel insights into their composition and functional significance. The identification of specific proteins and enrichment of distinct biological processes in each sample highlight the unique roles of the OF and eggs in sturgeon reproduction (Fig. 7).

## Methods

### Broodstock management, spawning and gamete collection

The Siberian sturgeons were maintained at the Department of Sturgeon Fish Breeding Inland Fisheries Institute in Pieczarki, Poland. The experiments were performed in the spawning season (May 2021) on five females (aged 9–14 years, body weight 16.5 ± 2.7 kg). The sampling was performed using the reproduction protocol previously described by Szczepkowski [61]. Lighting for 24 h/0 h provided photostimulation, and the water temperature in the tanks was 16 °C. Females were hormonally stimulated with two doses of 0.1 mg kg^− 1^ luteinizing hormone-releasing hormone agonist (LH-RHa) (Genscript, Piscataway, NJ, USA). The first injection was administered 36 h before planned sampling, and the second injection was given 24 h before sampling [62]. OF and eggs from the same female (*n* = 5) were collected using a catheter [63] into separate dry plastic containers for each female. OF was collected from the surface of the egg using a pipette, taking care to avoid any contamination with blood. For fertilization, semen was collected from hormonally stimulated males using a catheter [64]. Before each handling, the fish were anesthetized via immersion in MS-222 solution (150 mg L^− 1^; Sigma‒Aldrich, St. Louis., MO, USA). Ethics committee approval was not required for the experiments.

### Gamete management and fertilization

Each OF (*n* = 5) and egg (*n* = 5) sample was first aliquoted into cryotubes and frozen in liquid nitrogen. The remaining eggs were used for fertilization. Each portion of eggs (app. 1.5 kg in portion) was fertilized with pooled milt collected from three males. Sperm motility was greater than 89%. The milt was diluted to 1:100 (20 mL per 1.5 kg of eggs) and incubated with the eggs for 3 min. The eggs were double-washed with hatchery water. Adhesiveness was removed from the eggs by double-washing with a tannin solution at a 1:2000 dilution then again in hatchery water [65]. After de-adhesion, the eggs were placed in McDonald jars. Fertilization success was determined at the second cleavage (4 h postfertilization) and hatching (after 7 days of incubation at  15 °C) stages.

### Measurement of OF quality parameters

Osmolality was measured using a Minitübe Abfüll-u Labortechnik Löser apparatus (Tiefenbach, Germany). The protein concentration was measured by the Bradford method using a Coomassie Plus Kit (Thermo Scientific, Rockford, IL, USA) with bovine serum albumin as a standard. The pH of the OF was determined with a portable pH meter (HI9321 Hanna Instruments, USA).

### Protein extraction from eggs

Proteins from eggs (100 mg) were extracted in lysis buffer (400 µl; 8 M urea, 2% CHAPS, 30 mM Tris, pH 8.5) containing 0.5% protease inhibitor cocktail (Sigma‒Aldrich). Egg cells were homogenized for 4 × 30 s in 4 volumes of lysis buffer in a FastPrep-24 homogenizer and sonicated for 6 × 5 s on ice. The samples were incubated for 1 h on ice to facilitate protein extraction. Following incubation, the samples were centrifuged at 14 000×g for 15 min at 4 °C to separate the protein extract from the cellular debris. The protein concentration was measured using a Coomassie Plus Kit (Thermo Scientific) with bovine serum albumin as the standard. The protein extract (supernatant) was stored at -80 °C until analysis. The protein extracts were subjected to LC‒MS/MS and Western blot analyses.

### Liquid Chromatography–Mass Spectrometry

#### Protein digestion

Proteomic analysis was performed at the Mass Spectrometry Laboratory at the Institute of Biochemistry and Biophysics PAS, Warsaw.

Seventy micrograms of protein from each sample was digested according to the FASP protocol [66]. Cysteine groups were reduced by 1 h of incubation with 10 mM Tris (2-carboxyethyl) phosphine (TCEP) at 37 °C on a vortex. Protein solutions were transferred onto a Vivacon 30 kDa molecular weight cutoff filter (Sartorius Stedim). The filters were centrifuged at 14,500 × g for 15 min and washed with 200 µL of urea solution (8 M urea in 100 mM ammonium bicarbonate). Cysteines were alkylated via a 10-min incubation at room temperature with 25 mM methyl methanethiosulfonate (MMTS). The filters were washed three times with urea solution and 100 mM ammonium bicarbonate. After each addition, the samples were centrifuged until the cutoff filter was dry. Digestion was performed overnight using 2 µg of trypsin (Promega, WI, USA) at 37 °C (with an enzyme: protein ratio of 1:35). Peptides were eluted from spin filters by two 50 µL additions of 100 mM ammonium bicarbonate and one of 500 mM NaCl solution. The SpeedVac-dried samples were resuspended in 100 µL of 0.1% formic acid (FA) in water. Peptide concentrations were determined using a Pierce™ Quantitative Colorimetric Peptide Assay (Thermo Fisher Scientific).

#### Mass spectrometry

The samples were analyzed using an LC‒MS/MS system composed of an Evosep One HPLC System (Evosep Biosystems, Odense, Danemark) coupled to an Orbitrap Exploris 480 mass spectrometer (Thermo Scientific). One microgram of peptide solution was loaded onto an Evotips C18 disposable trap column as previously described [67]. Peptides were fractionated using an 88 min (15 samples per day) predefined Evosep gradient at a flow rate of 220 nl/min on an analytical column (Dr Maisch C18 AQ, 1.9 μm beads, 150 μm ID, 15 cm long, Evosep Biosystems). The following data-dependent acquisition parameters were used: the top 40 precursors were selected for MS2 analysis, and a collisional induced fragmentation NCE of 30%, spray voltage of 2.1 kV, funnel RF level of 40, and heated capillary temperature of 275 °C. Full MS scans covered the mass range of 300–1600 m/z with a resolution of 60,000, a maximum injection time set to Auto and a normalized AGC target to 300%. MS2 scans were acquired at a resolution of 15,000, an automatic maximum injection time and a standard AGC target. The ion isolation window was set to 1.6 m/z, the dynamic exclusion time was set to 20 s, and the minimum intensity threshold was set to 5e3.

#### Data analysis

The raw data files were preprocessed with Mascot Distiller (version 2.7, Matrixscience, London, U.K) and searched against the *Acipenser* protein database derived from NCBI (version from 2021.12.12, 78.501 sequences) supplemented with popular contaminants (cRAP database, 115 sequences). Mass tolerance was set individually after offline mass recalibration (typical values for peptide mass tolerance: 5 ppm; for fragments– 0.01 Da). Other parameters were set as follows: enzyme– trypsin, fixed modifications– methylthio (C), variable modifications– oxidation (M), and missed cleavages– 1. The data were incorporated into the in-house data analysis software Mscan (http://proteom.ibb.waw.pl/mscan/). The FDR was calculated with the target/decoy strategy and kept under 1%. To obtain protein abundance estimates, a normalized spectral counting approach was used. For each sample, emPAI values were calculated using the numbers of unique precursors normalized to the total number of precursors to account for loading differences. The comparison of the main components of the two sample types was based on the fold-change values obtained by dividing the median emPAI from each sample group. These resulting values were used to approximate which proteins predominated in each type of sample.

### Functional enrichment analysis of ovarian fluid and eggs

Gene Ontology (GO) annotations of identified proteins were obtained using the category ‘biological process’ (cut off at FDR = 0.05, ShinyGO v0.77: Gene Ontology Enrichment Analysis, http://bioinformatics.sdstate.edu/go/). The IPA (Qiagen, CA, US) of the identified proteins was used to interpret the identified proteins in the context of molecular and cellular functions and canonical pathways. The GI numbers of the identified proteins were matched to the UniProtKB database (www.UniProt.org). For protein–protein interaction network analysis, the differentially expressed proteins were analyzed using the Search Tool for the Retrieval of Interacting Genes/Proteins (STRING) database (Search Tool for the Retrieval of Interacting Genes, http://string-db.org/). The search for interactions was restricted to *Homo sapiens* protein pairs. The reliability of the interactions between proteins was assessed using a combined score (edge score).

### Western blot analysis

We used a Western blot procedure using stain-free gels (V3 stain-free workflow, Bio-Rad, Hercules, CA, USA) to validate the mass spectrometry results, as previously described [68], with some modifications. This method eliminates the need for the use of housekeeping proteins as loading controls for Western blots [69]. The expression of four proteins of interest, ALB, VTG2, FGB and FN1, was evaluated in the OF and eggs of sturgeon. The method of protein extraction from eggs was described above, and the OF was centrifuged at 3000 × g for 10 min at 4 °C. Equal amounts of protein (15 µg for FN1, 20 µg for VTG2 and ALB, 30 µg for FGB) were applied to Mini-Protean TGX Stain-Free 4–20% Gels (Bio-Rad). The quality of protein separation was checked after gel activation on a ChemiDoc instrument (Bio-Rad), and proteins were transferred to nitrocellulose membranes (0.22 μm) using a Mini Trans Biol Cell (Bio-Rad) in 20 mM Tris-HCl (pH 8.2), 150 mM glycine, and 10% methanol at 60 V for 90 min (4 °C). Nitrocellulose membranes were briefly rinsed in distilled water and blocked with 5% bovine serum albumin (Sigma‒Aldrich). The membranes were incubated overnight at 4 °C with primary polyclonal antibodies against ALB (1:1000), FGB (1:30000), FN1 (1:5000) and VTG2 (1:5000) (Supplementary Table S12). The membrane was rinsed to remove unbound primary antibodies and exposed to goat anti-rabbit antibodies (1:10000; Sigma‒Aldrich) linked to alkaline phosphatase. The products were visualized via incubation in a solution of alkaline phosphate buffer containing nitro blue tetrazolium (Sigma‒Aldrich) and 5-bromo-4-chloro-3-indolyl phosphate (Sigma‒Aldrich) in the dark. The staining was stopped with 0.2 M EDTA. Antibody-bound proteins were detected via enhanced chemiluminescence using a ChemiDoc imaging system (Bio-Rad). The optical density of the protein bands detected on the membranes and the intensity of the protein bands on the TGX Stain-Free gels were analyzed using Image Lab 6 software (Bio-Rad). The image of the gel acquired before its transfer was used as a control for equal protein loading between samples. The volume density of each target protein band was normalized to its respective total protein content, and the total protein band density was normalized to the total protein loaded into each lane using stain-free technology. The data are expressed in arbitrary units according to the manufacturer’s instructions (Bio-Rad) and Posch et al. [69]. To confirm the specificity of the antibodies used, protein bands corresponding to the detected bands were manually removed from the gels and prepared for digestion and protein identification using MALDI-TOF/TOF [70].

### Experimental design and statistical rationale

To evaluate the protein composition and differences between the proteomes of the OF and eggs, the present study performed five biological replicates using LC‒MS/MS analysis. The resulting MS/MS data were processed using Mscan software. The FDR thresholds for proteins were specified at 1%. To estimate the protein abundance, emPAI was calculated using unique precursors and normalized by total abundance. The differentially abundant proteins were calculated (IBB, Warsaw) (*p* < 0.05, fold change > 3). Gene Ontology (GO) enrichment, KEGG pathways, and IPA (Ingenuity Pathway Analysis) were used to examine the functional significance of the identified proteins. Protein–protein interaction network analysis was performed using the STRING database with a medium confidence score cutoff of 0.4. The expression of four identified proteins was confirmed by Western blotting using specific antibodies. All of the details are described above.

### Electronic supplementary material

Below is the link to the electronic supplementary material.

Supplementary Material 1

Supplementary Material 2

Supplementary Material 3

Supplementary Material 4

Supplementary Material 5

Supplementary Material 6

Supplementary Material 7

Supplementary Material 8

Supplementary Material 9

Supplementary Material 10

Supplementary Material 11

Supplementary Material 12

Supplementary Material 13

Supplementary Material 14

Supplementary Material 15

Supplementary Material 16

## Data Availability

The mass spectrometry proteomics data have been deposited in the ProteomeXchange Consortium via the PRIDE [71] partner repository with the dataset identifiers PXD044168 and 10.6019/PXD044168.

## Abbreviations

APPs: acute phase proteins
DAPs: differentially abundant proteins
ECM: extracellular matrix
emPAI: exponentially modified protein abundance index
EIFs: eukaryotic initiation factors
FA: formic acid
GO: Gene Ontology
HDL: high-density lipoprotein
IPA: Ingenuity Pathway Analysis
LH-RHa: luteinizing hormone releasing hormone agonist
OF: ovarian fluid
STRING: Search Tool for the Retrieval of Interacting Genes/Proteins
VLDL: very low density lipoprotein
