# Supplementary material for: Comparative proteomic analysis of the ovarian fluid and eggs of Siberian sturgeon
Source: BMC Genomics. 2024 May 7;25:451. doi: 10.1186/s12864-024-10309-y (PMC11077782; doi:10.1186/s12864-024-10309-y)
Supplement: Supplementary file 4 — Supplementary Material 4 [file 12864_2024_10309_MOESM4_ESM.pptx]

## Slide 1
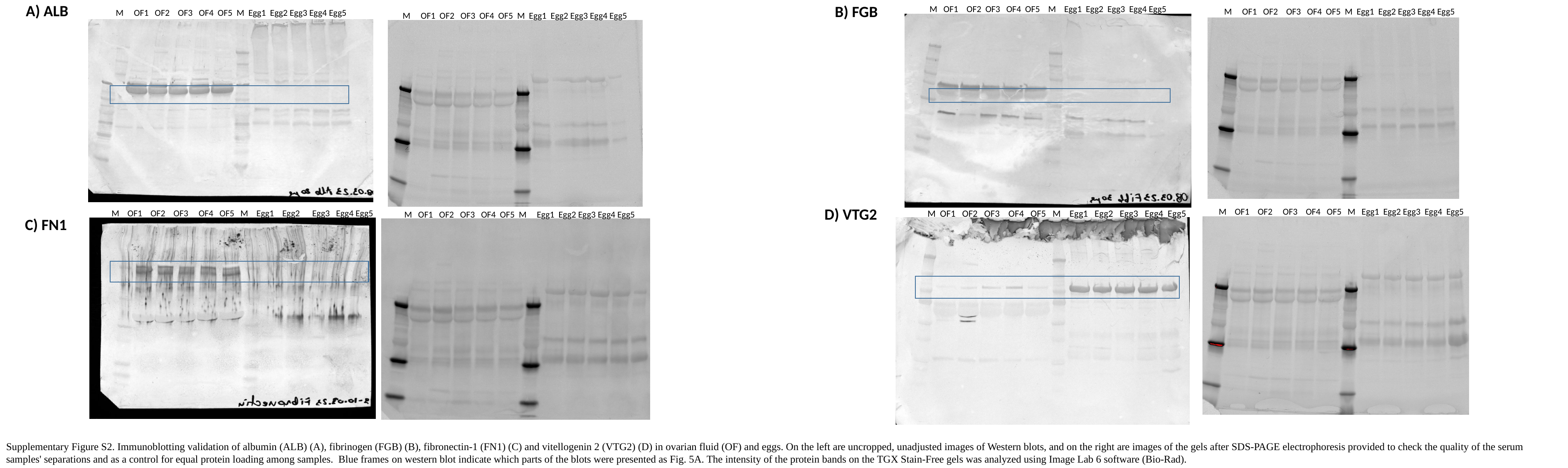

A) ALB
 M OF1 OF2 OF3 OF4 OF5 M Egg1 Egg2 Egg3 Egg4 Egg5
B) FGB
 M OF1 OF2 OF3 OF4 OF5 M Egg1 Egg2 Egg3 Egg4 Egg5
 M OF1 OF2 OF3 OF4 OF5 M Egg1 Egg2 Egg3 Egg4 Egg5
 M OF1 OF2 OF3 OF4 OF5 M Egg1 Egg2 Egg3 Egg4 Egg5
D) VTG2
M OF1 OF2 OF3 OF4 OF5 M Egg1 Egg2 Egg3 Egg4 Egg5
 M OF1 OF2 OF3 OF4 OF5 M Egg1 Egg2 Egg3 Egg4 Egg5
 M OF1 OF2 OF3 OF4 OF5 M Egg1 Egg2 Egg3 Egg4 Egg5
C) FN1
 M OF1 OF2 OF3 OF4 OF5 M Egg1 Egg2 Egg3 Egg4 Egg5
Supplementary Figure S2. Immunoblotting validation of albumin (ALB) (A), fibrinogen (FGB) (B), fibronectin-1 (FN1) (C) and vitellogenin 2 (VTG2) (D) in ovarian fluid (OF) and eggs. On the left are uncropped, unadjusted images of Western blots, and on the right are images of the gels after SDS-PAGE electrophoresis provided to check the quality of the serum samples' separations and as a control for equal protein loading among samples. Blue frames on western blot indicate which parts of the blots were presented as Fig. 5A. The intensity of the protein bands on the TGX Stain-Free gels was analyzed using Image Lab 6 software (Bio-Rad).
