# Supplementary material for: Comparative proteomic analysis of the ovarian fluid and eggs of Siberian sturgeon
Source: BMC Genomics. 2024 May 7;25:451. doi: 10.1186/s12864-024-10309-y (PMC11077782; doi:10.1186/s12864-024-10309-y)
Supplement: Supplementary file 9 — Supplementary Material 9 [file 12864_2024_10309_MOESM9_ESM.pptx]

## Slide 1
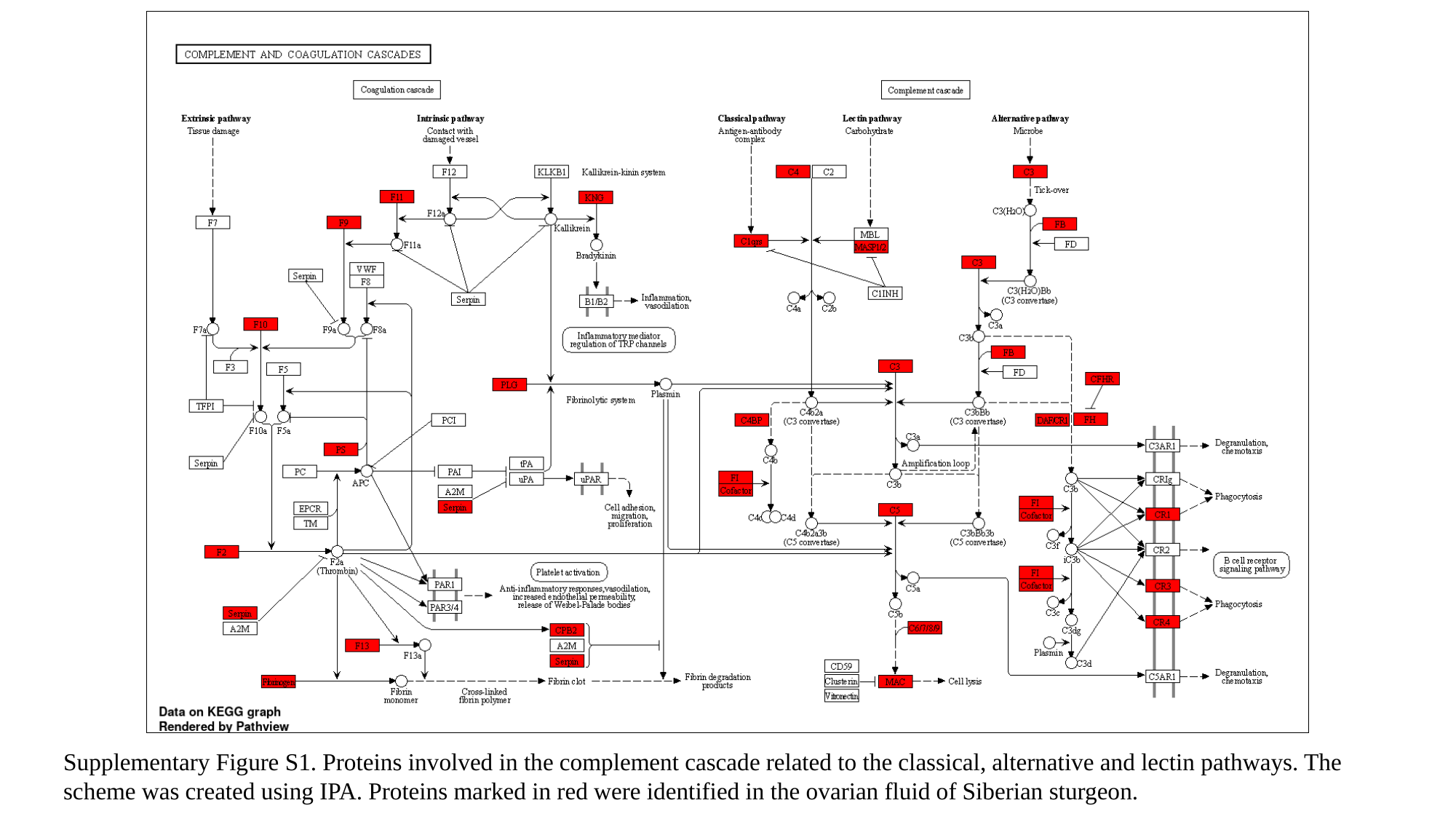

Supplementary Figure S1. Proteins involved in the complement cascade related to the classical, alternative and lectin pathways. The scheme was created using IPA. Proteins marked in red were identified in the ovarian fluid of Siberian sturgeon.
