# Supplementary material for: Comparative proteomic analysis of the ovarian fluid and eggs of Siberian sturgeon
Source: BMC Genomics. 2024 May 7;25:451. doi: 10.1186/s12864-024-10309-y (PMC11077782; doi:10.1186/s12864-024-10309-y)
Supplement: Supplementary file 10 — Supplementary Material 10 [file 12864_2024_10309_MOESM10_ESM.docx]

Supplementary Table S1. Characteristics of sturgeon ovarian fluid and egg quality.

| **No. Female** | **Age (years)** | **Weight (kg)** | **Protein concentration of ovarian fluid (mg mL^-1^)** | **Osmolality of ovarian fluid**  **(mOsm kg^-1^)** | **pH** | **Fertilization rate at the second cleavage** **(%)** | **Hatching rate (%)** |
| --- | --- | --- | --- | --- | --- | --- | --- |
| 1. | 14 | 20.4 | 2666.439 | 232 | 7.75 | 97.5 | 85 |
| 2. | 9 | 17.6 | 2709.502 | 251 | 7.72 | 99 | 62 |
| 3. | 14 | 16.4 | 2062.149 | 247 | 7.73 | 97 | 83 |
| 4. | 9 | 14.9 | 2767.484 | 227 | 7.74 | 100 | 76 |
| 5. | 14 | 13.3 | 2242.891 | 221 | 7.75 | 94 | 86 |
