# Supplementary material for: Comparative proteomic analysis of the ovarian fluid and eggs of Siberian sturgeon
Source: BMC Genomics. 2024 May 7;25:451. doi: 10.1186/s12864-024-10309-y (PMC11077782; doi:10.1186/s12864-024-10309-y)
Supplement: Supplementary file 12 — Supplementary Material 12 [file 12864_2024_10309_MOESM12_ESM.docx]

Supplementary Table S11. Identification of albumin, vitellogenin, fibrinogen and fibronectin from sturgeon ovarian fluid and eggs using MALDI-tof/tof.

| **Protein accession, description** | **Sequence coverage (%)** | **Mascot score** | **Calculated pI** | **Nominal mass (Da)** | **Precursor mass** | | **Peptide score** | **Peptide sequence** |
| --- | --- | --- | --- | --- | --- | --- | --- | --- |
|  |  |  |  |  | **observed** | **theoretical** |  |  |
| XP_033869101.2  serum albumin 2-like [*Acipenser ruthenus*] | *41* | 421 | 5.94 | 71.229 | 2181.0527  1460.9229  1043.5914  1391.7227 | 2179.9963  1459.9156  1042.5841  1390.7155 | 174  49  27  72 | R.YAQHGDQFLGSFLYEYSR.R  R.RHPELSIQVILR.I  K.FMLIQYTK.I  K.IMPQAPYDGLMR.V |
| XP_033858533.2  vitellogenin-like [*Acipenser ruthenus*] | *8* | 407 | 9.16 | 195.262 | 2334.2173  1131.7018  1578.8428  1512.7291  1759.8616 | 2333.2100  1130.6945  1577.8355  1511.7219  1758.8543 | 46  77  49  76  94 | R.GSLHYQFASELLQTPVQLFR.T  K.FLQLTQLLR.S  K.TLQPLHDLAADAASR.A  K.LFGQEVSFSDIDR.N  R.NSIENAVQTMTGPLER.Q |
| XP_033890063  fibrinogen alpha chain-like  *[Acipenser ruthenus*] | *17* | 328 | 5.34 | 86.514 | 1297.5155  2052.7929  1015.5472  3081.2266 | 1296.5769  2051.9371  1014.5862  3080.3523 | 38  35  43  119 | K.GYVGKDCDDIR.Q  K.MGFGSTNEQGQGELWLGNK.Y  K.YIHLLTQK.E  R.VELEDWEGHSTDAEYLVDVGPESEGYR.L |
| RXM99474.1  Fibronectin  [*Acipenser ruthenus*] | 5 | 146 | 5.32 | 270.667 | 1508.5834 | 1507.6151 | 52 | K.CDAIDQCQEPETP.I |
|  |  |  |  |  | 1741.7396 | 1740.8551 | 60 | R.nsfaevtglqpgtty.r |
|  |  |  |  |  | 2093.8857  1048.5242  2911.4587 | 2092.9037  1047.5364  2910.4631 | 52  27  76 | K.eylgaictctcfggqqgw.r  k.nsmtsrpv.r  K.taldsptgidfsevspnsltvhwlap.r |
