# Supplementary material for: Comparative proteomic analysis of the ovarian fluid and eggs of Siberian sturgeon
Source: BMC Genomics. 2024 May 7;25:451. doi: 10.1186/s12864-024-10309-y (PMC11077782; doi:10.1186/s12864-024-10309-y)
Supplement: Supplementary file 14 — Supplementary Material 14 [file 12864_2024_10309_MOESM14_ESM.pptx]

## Slide 1
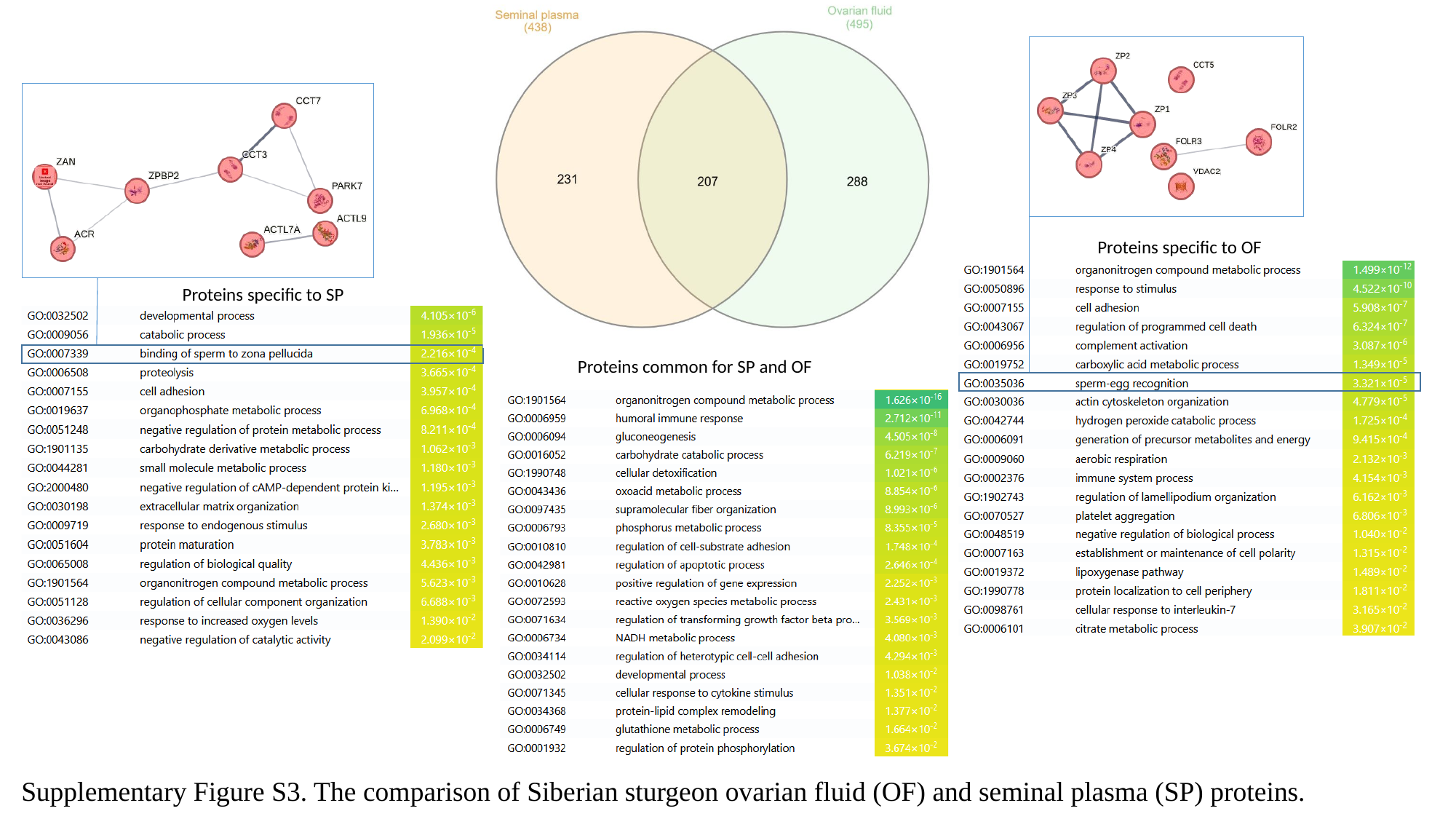

Proteins specific to OF
Proteins specific to SP
Proteins common for SP and OF
Supplementary Figure S3. The comparison of Siberian sturgeon ovarian fluid (OF) and seminal plasma (SP) proteins.
